# Distance-Based Queuing for Scalable and Reliable Linear Wireless Sensor Networks in Smart Cities

**DOI:** 10.3390/s24072023

**Published:** 2024-03-22

**Authors:** Iclia Villordo-Jimenez, Noé Torres-Cruz, Rolando Menchaca-Mendez, Mario E. Rivero-Angeles

**Affiliations:** 1 Unidad Profesional Interdisciplinaria en Ingeniería y Tecnologías Avanzadas, Instituto Politécnico Nacional, Mexico City 07340, Mexico; ntorresc@ipn.mx; 2 Centro de Investigación en Computación, Instituto Politécnico Nacional, Mexico City 07700, Mexico; rmen@cic.ipn.mx (R.M.-M.); mriveroa@ipn.mx (M.E.R.-A.)

**Keywords:** linear wireless sensor networks, queuing scheme, synchronized duty-cycled MAC, smart cities

## Abstract

The reliability and scalability of Linear Wireless Sensor Networks (LWSNs) are limited by the high packet loss probabilities (PLP) experienced by the packets generated at nodes far from the sink node. This is an important limitation in Smart City applications, where timely data collection is critical for decision making. Unfortunately, previous works have not addressed this problem and have only focused on improving the network’s overall performance. In this work, we propose a Distance-Based Queuing (DBQ) scheme that can be incorporated into MAC protocols for LWSNs to improve reliability and scalability without requiring extra local processing or additional signaling at the nodes. The DBQ scheme prioritizes the transmission of relay packets based on their hop distance to the sink node, ensuring that all packets experience the same PLP. To evaluate the effectiveness of our proposal, we developed an analytical model and conducted extensive discrete-event simulations. Our numerical results demonstrate that the DBQ scheme significantly improves the reliability and scalability of the network by achieving the same average PLP and throughput for all nodes, regardless of traffic intensities and network sizes.

## 1. Introduction

Wireless Sensor Networks (WSNs) have changed the way people interact with their environment by providing a cost-effective tool for collecting and delivering the information that supports today’s disruptive Internet of Things (IoT) and Smart City applications [1,2,3]. Specifically, Linear Wireless Sensor Networks (LWSNs), which are WSNs with linear or semi-linear topology, are well suited for monitoring a wide variety of systems, such as transportation networks or utility distribution systems (e.g., water, gas, and electricity), that also have linear or semi-linear structures [4,5,6]. These applications, where timely data collection and decision making are critical, require highly efficient and reliable sensing solutions to monitor the entire area of interest with adequate resolution [7].

For example, LWSNs will play a crucial role in Smart City applications, such as Smart Water Management (SWM) systems [8,9], where pipelines are sensed in a high number of points to measure water-quality parameters, the state of the infrastructure, and consumption. However, as a result of the large amount of traffic generated, the current supporting networks tend to be overloaded, limiting the reliability and scalability of the application [10,11]. Moreover, according to [12], in SWMs, as the number of nodes increases, the packet delivery ratio rapidly decreases, and, as a result, some areas become poorly sensed. This kind of problem has also been observed in other types of Smart City applications supported by LWSNs [1].

Previous works from multiple research groups, including ours, have proposed energy-efficient MAC protocols that take advantage of the fact that in LWSN data packets flow hop by hop from the node where the sensory data are generated towards the sink node located at one end of the network [13,14,15,16,17,18,19,20,21]. Despite the relevance of these works, they only focus on improving the overall network performance at the expense of the high packet loss probability (PLP) experienced by the data packets that have to travel through multiple relay nodes before reaching the sink. As a result, the network’s reliability and scalability are limited because the data collected by nodes located many hops away from the sink barely reach their destination [22,23]. While some previous works have analyzed the reliability of LWSNs in terms of failure, location, or coverage of the nodes [21,24,25], our focus is on improving the network’s reliability and scalability by modifying the MAC protocol, assuming that the nodes are active, and providing proper coverage. In this sense, our proposal is complementary to such works.

**Figure 1:**
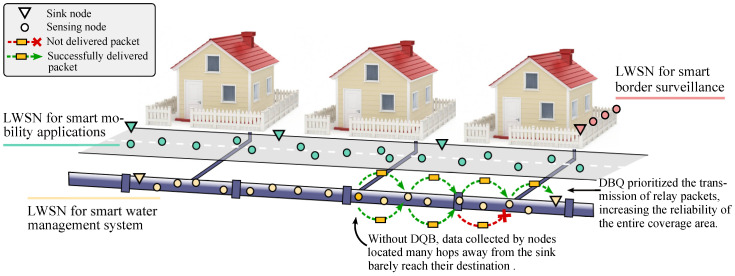
Contribution of the DBQ scheme to Smart City applications.

We introduce the Distance-Based Queuing (DBQ) scheme for LWSNs, which controls the proportion of local and relay packets transmitted by the nodes to provide the same average PLP and throughput for each node across the whole network. A set of analytical and simulation-based results show that DBQ effectively improves the network’s scalability in terms of its length while also increasing the reliability with which the entire coverage area is monitored. Moreover, the DBQ scheme achieves these goals without degrading the overall network performance or requiring extra signaling. In Figure 1 we illustrate the contribution of the DBQ scheme in the context of several Smart City applications, including SWM systems.

The rest of the paper is organized as follows. In Section 2 we review a representative sample of related work. Then, in Section 3, we describe our proposed queueing scheme. In Section 4, we briefly review the MAC protocol (HP-MAC) that we use as a case study to incorporate the DBQ Scheme. Based on the two previous sections, in Section 5 we develop a mathematical model to evaluate our proposal. In Section 6 we discuss on the robustness and the potential dynamic implementation of the DBQ Scheme. We discuss relevant numerical results and present our conclusions in Section 7 and Section 8, respectively.

## 2. Related Work

In [19], the authors present a comprehensive analytical method to evaluate Duty-Cycled, Pipelined-Forwarding (DCPF) MAC protocols for LWSNs. They present expressions to compute throughput, end-to-end packet delay, and energy consumption and apply their model to evaluate the performance of their proposed MAC protocol (PRI-MAC). Considering the same network topology, the authors of [15] propose an improvement to PRI-MAC by incorporating three algorithms that eliminate contention among nodes located within the same interference range; as a result, the performance in all the evaluated parameters improves significantly. In [13], the authors propose HP-MAC, which eliminates collisions by implementing distributed elections based on hash functions. The network nodes acquire transmission priorities, and only the node with the highest priority can transmit. As a result, significant gains in throughput and energy consumption are attained.

The authors of [18] also propose a DCPF MAC protocol (SA-MAC), where nodes selectively awaken depending on node density, traffic load conditions, and the state of the buffers of the receiving node. By implementing this strategy, energy consumption is reduced along the whole network; moreover, since the number of nodes contending for the channel decreases, the throughput and the end-to-end packet delay also improve. Similarly, in [14], an adaptive sleep/wake-up scheduling approach is proposed; however, in this case, the nodes’ decisions in each time slot are based on reinforcement learning.

The authors of [16] identified that DCPF networks may suffer from an energy-hole problem, where nodes near the sink node consume an excessive amount of energy. To address this problem, they propose the redundancy-based DCPF (RDCPF) protocol, which involves the deployment of redundant nodes in a LWSN. This approach results in a more uniform distribution of energy consumption throughout the network, thereby increasing the network’s survival time.

In [17], the authors propose a DCPF MAC protocol (JRAM) that, unlike previous works, assumes that a node has multiple chances to schedule data transmissions per cycle and, consequently, can transmit multiple data packets. As expected, the proposed strategy improves the network performance. The authors also developed a more general analytical model, which can be used to analyze previous DCPF MAC protocols as particular cases.

In [20,21], the authors analyze strictly linear WSNs, i.e., networks that consist of a single line of nodes (whereas in [13,14,15,16,17,18,19] the LWSN is assumed to be dense). The authors propose a routing algorithm that uses implicit acknowledgments to provide reliability and connectivity with energy efficiency, low latency, and fault tolerance.

As observed from the above-described works, multiple research groups have defined the features of MAC protocols that allow LWSNs to operate efficiently regarding energy consumption, throughput, and end-to-end packet delay. They have also developed rigorous analytical evaluation tools. However, these studies do not consider that the performance of different regions of the network can vary significantly. This is particularly true in areas far from the sink, where the nodes may experience poor performance, making the area barely sensed. Our main contribution is addressing this unsolved problem by developing a queue scheme that achieves a homogeneous performance of the LWSN in terms of throughput. We provide mathematical analysis and conduct extensive simulations to demonstrate the effectiveness of our proposal under various traffic conditions. Lastly, it is important to note that our scheme can complement any of the referred MAC protocols.

## 3. Distance-Based Queuing Scheme

**Figure 2:**
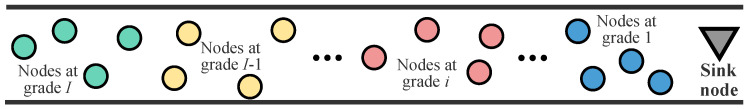
Dense LWSN, a topology with multiple nodes per grade.

The DBQ scheme is specifically suited for LWSNs that operate with synchronized Duty-Cycled, Pipelined-Forwarding (DCPF) MAC protocols [13,14,15,16,17,18,19]. In these networks, each node belongs to a grade i∈[1,I], where *i* is the node’s hop distance to the sink and *I* is the total number of grades in the network, i.e., the network length, as illustrated in Figure 2. More specifically, grade 1 is composed of the nodes that can reach the sink with a direct transmission, whereas grade 2 contains the nodes that can reach nodes in grade 1 with a direct transmission but not the sink. Packets generated at grade 2 require two hops to reach the sink. Nodes in grade *I* are the most remote nodes in the network. In a practical scenario, we can use the Grade Division and Pipelined Scheduling (GDPS) protocol [26] to compute the hop distance to the sink for each node in the network. This protocol can be applied during the network initialization.

Similar to [13,14,15,16,17,18,19], we consider a dense LWSN, in which more than one node can exist in each grade. Such a topology differs from strictly linear WSNs, e.g., [20,21], in which there exists only one line of nodes.

In LWSNs running a DCPF MAC protocol, the nodes follow a cycle that consists of (ξ+2) slots, which are arranged in the following order: a reception slot, a transmission slot, and ξ consecutive sleeping slots that are included to reduce energy consumption. The total cycle duration is $T_{c}=\left(\xi +2\right)T$ seconds, where *T* is the duration of one slot. The cycles for the nodes in the network are organized so that the transmission slots of nodes at grade i+1 are synchronized with the reception slots of nodes at grade *i* [13,19]. This synchronization allows data packets to be relayed from grade i+1 to grade *i* in a single slot.

To implement the DBQ scheme, each node requires two FIFO queues: one for serving local packets and another for serving relay packets. When a node gains access to the channel and has packets in both queues, it serves the queue for relay packets with the probability $p_{rel}$ and the queue for local packets with the probability $p_{loc}=1-p_{rel}$. If only one queue has packets, it will be served with a probability of one. While this way of serving transmission queues has been proposed before (e.g., [13]), the value of $p_{rel}$ was considered to be constant for each node in the network. However, in this work, we propose selecting $p_{rel}$ as a function of the nodes’ grade *i*. We denote as $p_{rel}^{i}$ the probability of selecting for transmission a packet from the relay buffer in a node at grade *i*.

To ensure that the PLP experienced by data packets is the same regardless of their originating node, the probabilities $p_{rel}^{i}$ have to be chosen in such a way that the sink receives roughly the same number of packets from nodes at every grade. We propose setting $p_{rel}^{i}$ to a specific value $p_{h}^{i}$, which has the effect of transmitting to the next grade the same number of packets from every grade j∈[i,I], namely, from every upstream grade and the current node’s grade. If all nodes in all grades follow this strategy, the nodes in grade 1 will deliver the same number of packets to the sink from every grade j∈[1,I] in the LWSN. This way, the DBQ scheme will homogenize the PLP performance across the entire network, as illustrated in Figure 2.

To determine the values of $p_{h}^{i}$, observe that homogenizing the PLP is equivalent to homogenizing the throughput. In order to do this, we define $S_{r}^{i}$ as the relay throughput that is received in the relay buffer of a node at grade *i*, in packets per cycle, from nodes in grade i+1. Similarly, we define $S_{l}^{i}$ as the locally generated throughput that is queued in a node at grade *i*. Note that these variables depend on $p_{rel}^{i}$ because increasing this probability has the effect of transmitting more relay packets than local ones, which in turn increases $S_{r}^{i}$ and decreases $S_{l}^{i}$ simultaneously.

**Figure 3:**
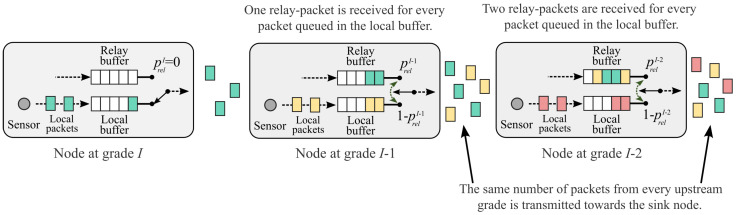
Homogeneous throughput achieved by the DBQ scheme.

To achieve a homogeneous throughput, nodes at grade I−1 (which are the most distant nodes from the sink that relay packets) should receive, on average, the same number of packets from grade *I* as the number of packets they queue in their local buffers. As a result, the nodes at grade I−1 transmit the same number of packets from grades *I* and I−1 to grade I−2. Similarly, the nodes at grade I−2 should receive, on average, two relay packets from grade I−1 for every packet queued in its local buffer so that nodes in grade I−2 can transmit the same number of packets from grades *I*, I−1, and I−2 to grade I−3. In general, to make sure that the data packets experience homogeneous throughput across the entire network, a node at grade *i* must receive, on average, I−i relay packets for every packet queued in its local buffer. This means that $S_{r}^{i}$ should be I−i times larger than $S_{l}^{i}$. This concept is illustrated in Figure 3.

In summary, the throughput per grade is homogeneous across the network if $S_{r}^{i}=\left(I-i\right)S_{l}^{i}$, for i∈[1,I−1]; i.e., if $S_{r}^{i}-\left(I-i\right)S_{l}^{i}$=0, for i∈[1,I−1]. Consequently, the problem of calculating the value of $p_{h}^{i}$ can be restated as finding the root of

(1) $$f\left(p_{rel}^{i}\right)=S_{r}^{i}-\left(I-i\right)S_{l}^{i},\;\;1\leq i\leq I-1;$$

which is a function of $p_{rel}^{i}\in \left[0,1\right]$ and represents the difference between the desired proportions of local and relay throughput.

## 4. HP-MAC Overview

Since the DBQ scheme is intended to be used with DCPF MAC protocols for LWSNs, we select, as a case study, the collision-free protocol known as Hash-based with Packet-prioritization MAC (HP-MAC) [13].

**Figure 4:**
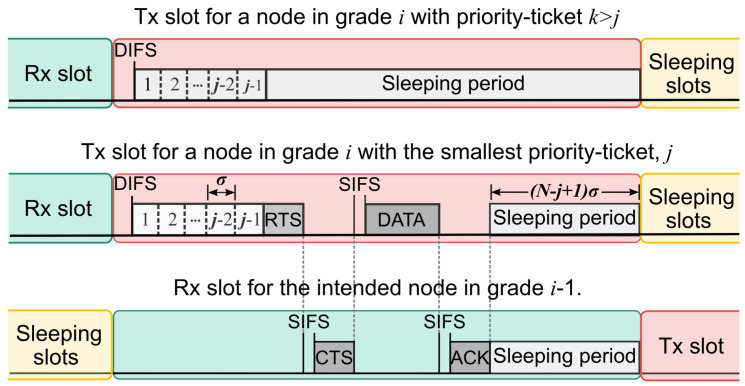
Slots structure and contention process in HP-MAC.

In HP-MAC, nodes have two queues of a length *K* to serve local and relay packets independently. At the beginning of a transmission slot, every node in the same grade, with queued packets, computes the same set of *N* priority tickets (where *N* is the number of nodes in a grade). This way, nodes acquire a unique priority transmission number in [1,N], which is used to control access to the channel. A node with priority *k* listens to the channel during k−1 mini-slots of duration σ. If it does not detect any transmission, it initiates an RTS-CTS-DATA-ACK interchange with a node in the immediate inferior grade. Otherwise, it goes to sleep as soon as a transmission is detected because a node with a higher priority j<k has accessed the channel. Figure 4 illustrates the frame structure of the contention process from the point of view of a node that detects a busy channel, the node that wins the contention, and the intended receiver node in the next grade.

In HP-MAC, the probability of transmitting a packet in a cycle, for a node in grade *i* with queued packets, denoted by $p_{t}\left(i\right)$, equals

(2) $$p_{t}\left(i\right)=\frac{1-p_{e,e}{\left(i\right)}^{N}}{N\left(1-p_{e,e}\left(i\right)\right)},$$

where $p_{e,e}\left(i\right)$ is the probability that none of the queues has packets at the beginning of a transmission slot. The probability of receiving a packet during a cycle, denoted by $p_{r}\left(i\right)$, is

(3) $$p_{r}\left(i\right)=p_{t}\left(i+1\right)\left[1-p_{e,e}\left(i+1\right)\right],\;\;i<I,$$

and $p_{r}\left(I\right)=0$, since nodes in grade *I* do not relay packets.

To obtain $p_{e,e}\left(i\right)$, one can solve a Discrete-Time Markov Chain (DTMC) for every grade *i*, whose steady-state solution is denoted by $\pi_{m,u}^{i}$, for m,n∈[0,K], and represents the steady-state probabilities of having *m* local and *u* relay packets in the corresponding queues. This DTMC depends on the probability of generating a local packet during a cycle (*a*) and the probability of selecting a packet from the relay queue ($p_{rel}^{i}$). The DTMC definition, its solution, and its relation with (2) and (3) are described in detail in [13], Section IV.B.

## 5. Analytical Model for the DBQ Scheme

In this section, we develop expressions to calculate the PLP and throughput for nodes in grade i∈[1,I], in terms of $p_{r}\left(i\right)$, $p_{t}\left(i\right)$, and $\pi_{m,u}^{i}$, considering an HP-MAC network where nodes implement the DBQ scheme. Then, we propose a method to obtain $p_{h}^{i}$, for i∈[1:I]; i.e., the values of $p_{rel}^{i}$ that homogenize the PLP and throughput across the whole network.

Our analytical model is based on the assumption that there are *I* grades, each consisting of *N* nodes. We also assume that local packets arrive at every node with a probability equal to *a* during a cycle. It is important to note that *a* is a measure of traffic intensity, and we consider it to be a constant for the steady-state analysis.

The expected number of successfully transmitted packets per cycle from grade i+1 to grade *i* is $Np_{t}\left(i+1\right)\left[1-p_{e,e}\left(i+1\right)\right]$; hence, the throughput from grade i+1 to grade *i* is, in general,

(4) $$\begin{array}{c} S_{r}^{i}=\frac{1}{T_{c}}Np_{t}\left(i+1\right)\left[1-p_{e,e}\left(i+1\right)\right]\left[1-b_{r}^{i}\right],1\leq i\leq I-1, \end{array}$$

where $b_{r}^{i}$ is the blocking probability (the probability of dropping an incoming packet) in the relay queue of a node in grade *i*, which can be expressed as

(5) $$b_{r}^{i}=\sum_{u=0}^{K}\pi_{K,u}^{i}.$$

In particular, the throughput from grade 1 to grade 0, $S_{r}^{0}$, represents the network-wide throughput that is delivered to the sink node; therefore, we also denote it as $S_{sink}$. Since we assume that the receiving queue in the sink node is infinite, we obtain

(6) $$\begin{array}{c} S_{sink}=S_{r}^{0}=\frac{1}{T_{c}}Np_{t}\left(1\right)\left[1-p_{e,e}\left(1\right)\right]. \end{array}$$

In addition, the throughput generated in a node’s local queue is defined as

(7) $$S_{l}^{i}=\frac{1}{T_{c}}N\times a\left[1-b_{l}^{i}\right],$$

where $b_{l}^{i}$ is the blocking probability in the local queue for a node in grade *i* and is given by

(8) $$b_{l}^{i}=\sum_{m=0}^{K}\pi_{m,K}^{i}.$$

Given that a packet generated in grade *i* has relayed through grades i−1,i−2,...,1 until reaching the sink, the throughput from grade *i*, which reaches the sink node, denoted by $S_{sink}^{i}$, can be written as

(9) $$S_{sink}^{i}=\frac{Na}{T_{c}}\left[1-b_{l}^{i}\right]\prod_{j=1}^{i-1}\left[1-b_{r}^{j}\right].$$

We can derive an alternative expression for calculating $S_{sink}$ from (9).

(10) $$\begin{array}{c} S_{sink}=\sum_{i=1}^{I}S_{sink}^{i}. \end{array}$$

Since new packets are generated in grade *i* at rate $\frac{N\;\times \;a}{T_{c}}$; we can compute the probability of losing a packet generated in grade *i*, denoted by $p_{lp}\left(i\right)$, as

(11) $$p_{lp}\left(i\right)=1-\frac{T_{c}S_{sink}^{i}}{N\times a}.$$

Now, we are ready to derive an expression for $f(p_{rel}^{i})$ (see (1), (4), and (7)) as follows.

(12) $$\begin{array}{cc} f(p_{rel}^{i})= & \frac{1}{T_{c}}Np_{t}\left(i+1\right)\left[1-p_{e,e}\left(i+1\right)\right]\left[1-b_{r}^{i}\right]\\ \; & -\left(I-i\right)\frac{1}{T_{c}}N\times a\left[1-b_{l}^{i}\right],\;\;i\in \left[1,I-1\right]; \end{array}$$

where (12) can be simplified to obtain (13).

(13) $$\begin{array}{c} f\left(p_{rel}^{i}\right)=p_{t}\left(i+1\right)\left[1-p_{e,e}\left(i+1\right)\right]\left[1-b_{r}^{i}\right]-\left(I-i\right)a\left[1-b_{l}^{i}\right],\;\;i\in \left[1,I-1\right]. \end{array}$$

Even though, the dependence on $p_{rel}^{i}$ is not explicit on the right side of (13); it does exist because $b_{r}^{i}$ and $b_{l}^{i}$ are obtained in terms of the DTMC solution, which directly depends on $p_{rel}^{i}$. The remaining parameters in (13) are constants or depend on $p_{h}^{i+1}$, which must be previously calculated. Lastly, $p_{h}^{I}$ must be set to 0 since nodes in grade *I* do not relay packets.

As we have mentioned, the roots of $f(p_{rel}^{i})$ are the values of $p_{h}^{i}$ that homogenize the PLP across the network. To find these roots, we note that as $p_{rel}^{i}$ increases, $b_{r}^{i}$ decreases, and $b_{l}^{i}$ increases because the priority of the relay packets over the local packets increases; therefore, $f(p_{rel}^{i})$ is a monotonically increasing function with at most one root.

To determine whether $f(p_{rel}^{i})$ has one root or none, first, we analyze the scenario where the packet arrival rate, considering both local and relay traffic, is large enough to produce a blocking probability larger than zero in at least one of the buffers. In this scenario, which we refer to as high-traffic conditions, if $p_{rel}^{i}\to 0$, then $b_{r}^{i}\to 1$ because relay packets are not allowed to be transmitted, and $b_{l}^{i}$ is less than 1. Consequently, $f(p_{rel}^{i})$ is negative, as we can observe from (13). Analogously, we can conclude that $f(p_{rel}^{i})$ is positive if $p_{rel}^{i}\to 1$. Based on these arguments, we can conclude that $f(p_{rel}^{i})$ has one root under high-traffic conditions, which can be found using standard numerical methods. To evaluate every instance of $f(p_{rel}^{i})$, as required to implement the numerical method, we need to solve the system of equations comprised (2), (3), and the above-mentioned DTMC, for i∈[1,I].

On the other hand, when the packet arrival rate is very low, $b_{r}^{i}$ and $b_{l}^{i}$ tend to zero, as well as the corresponding blocking probabilities for nodes in grades larger than *i*. Under these low-traffic conditions, the number of received relay packets in grade *i* is I−i times larger than the number of admitted local packets, which means that homogeneous PLP conditions hold for a wide range of values of $p_{rel}^{i}$ and there is no need to find the root of $f(p_{rel}^{i})$. Moreover, since under low-traffic conditions $f(p_{rel}^{i})$ tends to zero, finding its root can be limited by the resolution of the implementation of the numerical method. Therefore, we can simply select $p_{h}^{i}$ as the ratio of the relay throughput to the total throughput, i.e.,

(14) $$\begin{array}{c} p_{h}^{i}=\frac{I-i}{I-i+1}. \end{array}$$

Lastly, to determine whether a grade *i* is under high- or low-traffic conditions, we define a parameter δ, which represents the largest difference between the desired proportions of relay and local throughput that is considered acceptable in terms of homogeneous PLP conditions. If the range of $f(p_{rel}^{i})$, given by $R_{i}=f\left(1\right)-f\left(0\right)$, is smaller than δ, we consider that the LWSN is under low-traffic conditions. Algorithm 1 summarizes the above-described method.

According to Algorithm 1, the computation time of the proposed method is proportional to the network length *I* and to the complexity of the method to find $p_{h}^{i}$. For the case of the bisection method, the complexity is $O(log\frac{1}{\epsilon })$, where ϵ is the maximum error allowed in the solution.
**Algorithm 1** Selection of the relay probabilities, $p_{h}^{i}$.$p_{h}^{I}\leftarrow$ 0**for**i=I−1→1**do**    Calculate $R_{i}=f\left(1\right)-f\left(0\right)$    **if** $R_{i}<\delta$ **then**        $p_{h}^{i}\leftarrow \frac{I-i}{I-i+1}$    **else**        Find the root of $f(p_{rel}^{i})$, $p_{h}^{i}$    **end if****end for**

## 6. Robustness and Dynamic Implementation of the DBQ Scheme

The solution presented in the previous section to find $p_{h}^{i}$ has been developed for the steady state, which means that it is valid for a constant value of traffic-intensity, *a*. Under this assumption, the implementation of the DBQ scheme does not require extra signaling, as we have mentioned above, since nodes can be set with $p_{h}^{i}$ during the network-initialization stage. However, our method can also be used considering time-varying traffic. Under such dynamic conditions, it is necessary to continuously measure *a* and to recalculate $p_{h}^{i}$ if significant variations of *a* are detected. Since battery and processing capacity are very limited resources in the sensing nodes, the described dynamic recalculation of $p_{h}^{i}$ must be carried on remote servers, hence, requiring a bidirectional interchange of signaling between the servers and the sensing nodes.

Though such a solution may demand more resources from the LWSN (to the detriment of its lifetime), we consider that implementing it is feasible because the features of a particular application are known in advance, and, therefore, a proper network design may avoid extreme traffic variations. Moreover, the dynamic estimation of $p_{h}^{i}$ increases the robustness of the DBQ scheme.

The previous arguments are valid not only in terms of *a* but also in terms of any other phenomenon that directly or indirectly affects the traffic intensity. As another example, notice that nodes failure decrease the number of nodes per grade, *N*, which, in turn, increases the average number of relay packets that a node must process. Therefore, in the case of node failure, $p_{h}^{i}$ must be recalculated (the dependence on *N* is explicit in our model), and the solution is feasible, if failures are maintained under control.

In general, we can affirm that the dynamic implementation of the DBQ scheme increases its robustness, given a proper network design.

## 7. Numerical Results and Discussion

To evaluate the performance gains achieved by the DBQ scheme, we consider an LWSN with I=7, N=10, K=7, ξ=18, and T=111 ms, unless otherwise specified. These values were selected to evaluate the network performance under different traffic conditions and considering realistic settings. For example, in [9,27,28], experimental tests were conducted with tens of sensing nodes and at most three hops along the network. Additionally, in [29], the authors report a real-world WSN implemented with tens of sensing nodes per sink node. We propose setting the value of δ to 0.001, meaning that we consider that the network is operating under homogeneous-PLP conditions if the maximum difference between relay and local throughputs is less than or equal to 0.1% (given that the maximum throughput is 1 packet/cycle). As shown in Algorithm 1, we set $p_{h}^{I}=0$. Then, we estimate $p_{h}^{i}$ for i∈[1,I−1]. When high-traffic conditions are detected, we obtain the root of $f(p_{rel}^{i})$ through the bisection method with an error of $\epsilon =1\times 10^{-4}$.

Table 1 shows a set of numerical results for $p_{h}^{i}$ under different values of *a*. The yellow cells indicate the grades that operate under high-traffic conditions. As expected, these cases occur in the grades closest to the sink, and the number of grades in these conditions increases as the value of *a* increases. We observe that, for a given value of *a*, the value of $p_{h}^{i}$ increases as the number of hops required to reach the sink decreases because the aggregated relay traffic is significantly larger than the local traffic. As a result, the priorities of the relay queues in the nodes close to the sink have to be higher than the priorities of the local ones. It is interesting to note that, for a given grade *i*, the range of values of $p_{h}^{i}$, as a function of *a*, is small, which means that the estimation of $p_{h}^{i}$ must be very precise.

**Figure 5:**
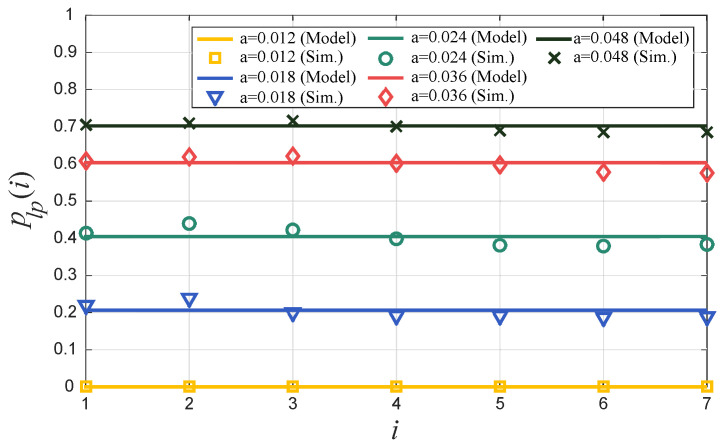
Analytical and simulation-based results for the packet loss probability $p_{lp}\left(i\right)$ at grade *i* for different values of traffic intensity *a*.

After finding the values of the probabilities $p_{h}^{i}$, we set the priorities of the queues with such values. We refer to this version of HP-MAC as HP-MAC/DBQ. Figure 5 shows the resulting values of $p_{lp}\left(i\right)$ for different traffic intensities, *a*. We observe that the DBQ mechanism effectively homogenizes the LWSN’s PLP under a wide range of traffic intensities, validating the effectiveness of our method. The homogeneous performance will allow the LWSN to monitor the whole area of interest with the same quality and resolution, thus preventing the emergence of poorly covered areas. Figure 5 also shows the results of extensive discrete event simulations (we executed 1 $\times \;10^{5}$ cycles for every set of input parameter values), which confirm the predictions of the analytical model.

**Figure 6:**
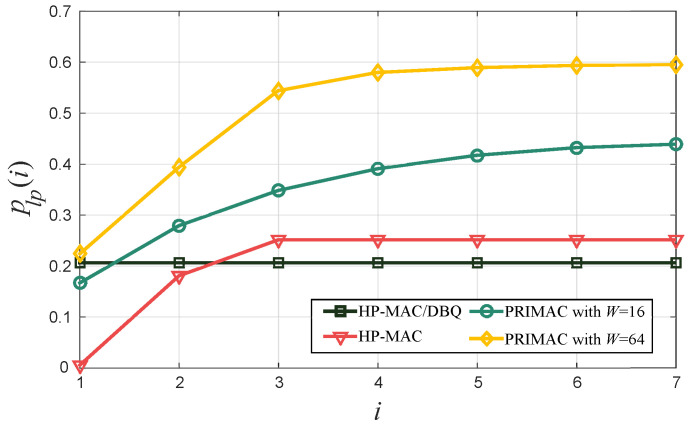
Packet loss probability $p_{lp}\left(i\right)$ per grade *i*.

Figure 6 shows the PLP per grade *i* attained by our proposal, HP-MAC/DBQ, and two other state-of-the-art protocols, HP-MAC [13] and PRIMAC [19]. Since the performance of PRIMAC depends on the size *W* of the contention window, we consider two representative values, 16 and 64. As expected, the PLP for HP-MAC and PRIMAC degrades rapidly as the hop distance to the sink increases because data packets can be lost at each hop while competing for network resources with the locally generated data packets. On the other hand, HP-MAC/DBQ outperforms the other protocols for all grades except those closest to the sink, which is the result of assigning more resources to the packets generated upstream.

**Figure 7:**
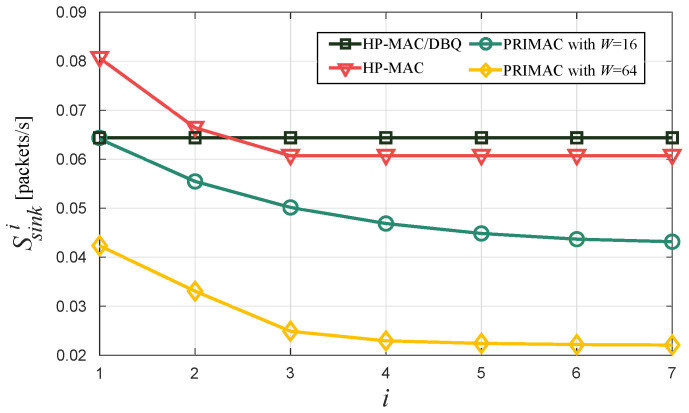
Throughput $S_{sink}^{i}$ per grade *i*.

Similarly, Figure 7 presents the throughput attained by the three protocols. Again, we observe that only HP-MAC/DBQ exhibits a homogeneous throughput across the network and, in general, outperforms the remaining protocols.

**Figure 8:**
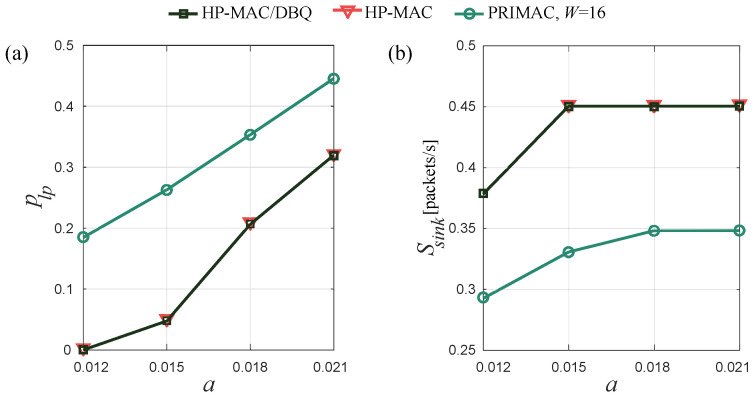
Network-wide (**a**) average PLP and (**b**) throughput.

In Figure 8a,b, we present the network-wide average PLP and throughput attained by the protocols as *a* increases, respectively. The figures show that the overall performance exhibited by HP-MAC/DBQ is equivalent to that of HP-MAC, indicating that the proposed DBQ scheme does not have a negative impact on the network-wide performance of HP-MAC. This is mainly due to two reasons. Firstly, the DBQ scheme does not interfere with the underlying contention avoidance scheme and does not require additional signaling. Secondly, by solving (13), the DBQ scheme assigns the appropriate amount of network resources to data packets, ensuring that all packets have roughly the same probability of reaching the sink, regardless of their originating grade. The figure also shows that both HP-MAC and HP-MAC/DBQ outperform PRIMAC.

**Figure 9:**
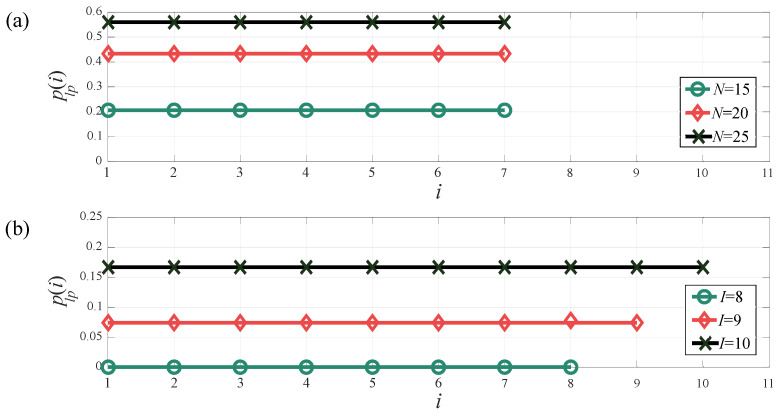
Packet loss probability per grade (**a**) as a function of *N*, for I=7, and (**b**) as a function of *I*, for N=10.

In Figure 9a,b, we present the PLP performance of the LWSN in terms of *I* and *N*, respectively. In these evaluations, we have assumed a value of a=0.012. We notice that as an inherent effect of increasing the network size, the PLP increases as *N* or *I* increases. It is important to note that despite the high-intensity traffic, the uniform performance remains unaffected, which means that $p_{lp}\left(i\right)$ remains constant throughout the network for any given input parameters.

As a result of having a homogeneous PLP, the throughput per grade that reaches the sink, $S_{sink}^{i}$, is also homogeneous; i.e., it is the same for any grade *i*. To illustrate this behavior, we present in Table 2 and Table 3 the value of $S_{sink}^{i}$ as well as the value of the network-wide throughput, $S_{sink}$, as a function of *N* and *I*. We observe that as *N* increases, both $S_{sink}^{i}$ and $S_{sink}$ decrease because in HP-MAC, the frame duration increases with *N*, hence, reducing the throughput that the network can deliver. On the other hand, when *I* increases, the frame duration remains constant, resulting in an increase in $S_{sink}$ because more packets are generated in the network. Nevertheless, since the network capacity must be shared among a larger number of grades, $S_{sink}^{i}$ decreases. From this analysis, we can conclude that the network-wide throughput is affected by the MAC protocol features (HP-MAC in this case) but not by the incorporation of the DBQ scheme.

**Figure 10:**
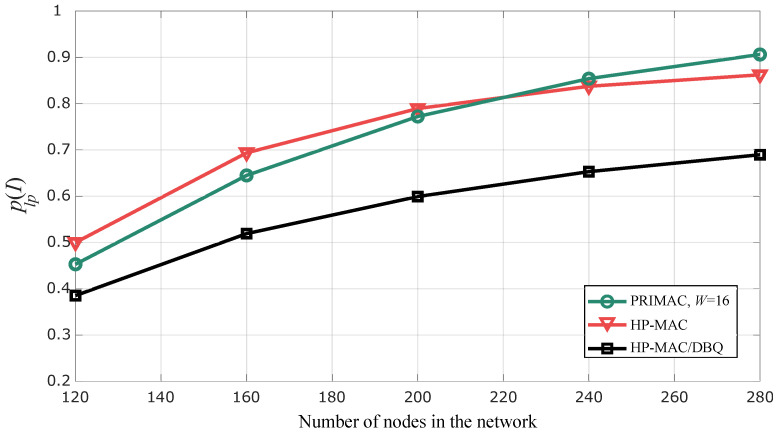
Packet loss probability for remote nodes (grade *I*) in terms of the number of nodes in the network.

Lastly, to illustrate how our proposal improves the reliability of the network, we show the PLP for the most remote nodes of the network, $p_{lp}\left(I\right)$, in Figure 10. For these results, we consider a=0.012 and I=8. We observe that, for 120 nodes, all the protocols exhibit similar performances. However, as the total number of nodes increases in the order of hundreds (we increase *N* from 15 to 35), $p_{lp}\left(I\right)$ for PRIMAC and HP-MAC tends toward 1, which means that the sensing of the most remote regions of the network becomes very unreliable. By contrast, the implementation of the DBQ scheme maintains a more reliable sensing of the whole network under a wider range of possible scenarios. Evidently, these reliability improvements are limited by the network capacity.

## 8. Conclusions

In previous DCPF MAC protocols for LWSN, the performance experienced by the data packets strongly depends on the hop distance between the source nodes and the sink, which severely limits the network scalability in terms of its length because packets generated many hops away from the sink barely reach their destination. This is unacceptable from the applications’ point of view because the entire area of interest covered by the LWSN is equally important and should be monitored with the same quality. The proposed DBQ scheme solves this problem by improving the reliability and scalability of an LWSN running a DCPF MAC protocol without requiring additional signaling or local processing at the nodes. Our analytical and simulation-based results show that DBQ is effective under a wide range of traffic conditions and network sizes, making it a viable solution for LWSNs.

Our results also validate the accuracy of the proposed analytical model and demonstrate that it is an effective tool for determining the probabilities of serving the relay queues that achieve homogeneous performance across the whole LWSN. This is true even when the network performance is highly sensitive to these probabilities.

Future work includes evaluating the effectiveness of the proposed scheme under dynamic traffic conditions and developing solutions that jointly homogenize packet loss probability and end-to-end delay.

## Figures and Tables

**Table 1:** Probability of selecting the relay buffer that homogenizes the network performance, $p_{h}^{i}$, for different grades *i* and traffic intensities *a*.

*a*	Grade *i*						
	1	2	3	4	5	6	7
**0.012**	0.857	0.833	0.800	0.750	0.667	0.500	0
**0.018**	0.910	0.899	0.800	0.750	0.667	0.500	0
**0.024**	0.928	0.883	0.829	0.750	0.667	0.500	0
**0.036**	0.938	0.895	0.842	0.768	0.699	0.500	0
**0.048**	0.941	0.899	0.846	0.777	0.672	0.500	0

**Table 2:** Per grade and network-wide throughput as a function of *N*, for I=7.

*N*	$S_{\mathrm{sink}}^{i}$	$S_{\mathrm{sink}}$
**15**	0.0616	0.4312
**20**	0.0590	0.4130
**25**	0.0567	0.3969

**Table 3:** Per grade and network-wide throughput as a function of *I*, for N=10.

*I*	$S_{\mathrm{sink}}^{i}$	$S_{\mathrm{sink}}$
**8**	0.0541	0.4328
**9**	0.0501	0.4509
**10**	0.0450	0.4500

## Data Availability

The original contributions presented in this study are included in the article; further inquiries can be directed to the corresponding author.

## Abbreviations

*a*	Probability of generating a local packet during a cycle;
$f(p_{rel}^{i})$	Difference between the desired proportions of local and relay throughput at grade *i*;
*I*	Number of grades in the network (network length);
*K*	Buffers’ length (in number of packets);
*N*	Number of nodes per grade;
$p_{h}^{i}$	Value of $p_{rel}^{i}$ that homogenizes the sent throughput toward grade i+1;
$p_{lp}\left(i\right)$	Probability of losing a packet generated in grade *i*;
$p_{rel}^{i}$	Probability of selecting for transmission a packet from the relay buffer at grade *i*;
$S_{l}^{i}$	Locally generated throughput that is queued in a node at grade *i*;
$S_{r}^{i}$	Relay throughput from grade i+1 that is received in a node at grade *i*;
$S_{sink}$	Network-wide throughput that is delivered to the sink node;
$S_{sink}^{i}$	Throughput from grade *i* that reaches the sink node;
*T*	Slot duration;
ξ	Number of sleeping slots during a cycle.
